# SARS-CoV-2 N501Y Introductions and Transmissions in Switzerland from Beginning of October 2020 to February 2021—Implementation of Swiss-Wide Diagnostic Screening and Whole Genome Sequencing

**DOI:** 10.3390/microorganisms9040677

**Published:** 2021-03-25

**Authors:** Ana Rita Goncalves Cabecinhas, Tim Roloff, Madlen Stange, Claire Bertelli, Michael Huber, Alban Ramette, Chaoran Chen, Sarah Nadeau, Yannick Gerth, Sabine Yerly, Onya Opota, Trestan Pillonel, Tobias Schuster, Cesar M. J. A. Metzger, Jonas Sieber, Michael Bel, Nadia Wohlwend, Christian Baumann, Michel C. Koch, Pascal Bittel, Karoline Leuzinger, Myrta Brunner, Franziska Suter-Riniker, Livia Berlinger, Kirstine K. Søgaard, Christiane Beckmann, Christoph Noppen, Maurice Redondo, Ingrid Steffen, Helena M. B. Seth-Smith, Alfredo Mari, Reto Lienhard, Martin Risch, Oliver Nolte, Isabella Eckerle, Gladys Martinetti Lucchini, Emma B. Hodcroft, Richard A. Neher, Tanja Stadler, Hans H. Hirsch, Stephen L. Leib, Lorenz Risch, Laurent Kaiser, Alexandra Trkola, Gilbert Greub, Adrian Egli

**Affiliations:** 1 Laboratory of Virology, University Hospital Geneva, 1205 Geneva, Switzerland; AnaRita.GoncalvesCabecinhas@hcuge.ch (A.R.G.C.); Sabine.Yerly@hcuge.ch (S.Y.); Isabella.Eckerle@hcuge.ch (I.E.); Laurent.kaiser@hcuge.ch (L.K.); 2 Center for Emerging Viral Diseases, University Hospital Geneva, 1205 Geneva, Switzerland; 3 Applied Microbiology Research, Department of Biomedicine, University of Basel, 4056 Basel, Switzerland; tim.roloff@ubs.ch (T.R.); madlen.stange@usb.ch (M.S.); myrta.brunner@unibas.ch (M.B.); kirstinekobberoee.soegaard@usb.ch (K.K.S.); Helena.Seth-Smith@usb.ch (H.M.B.S.-S.); alfredo.mari@unibas.ch (A.M.); 4 Clinical Bacteriology and Mycology, University Hospital Basel & University of Basel, 4031 Basel, Switzerland; 5 Swiss Institute for Bioinformatics (SIB), 1015 Lausanne, Switzerland; richard.neher@unibas.ch (R.A.N.); tanja.stadler@bsse.ethz.ch (T.S.); 6 Institute of Microbiology, Lausanne University Hospital and University of Lausanne, 1011 Lausanne, Switzerland; claire.bertelli@chuv.ch (C.B.); onya.opota@chuv.ch (O.O.); trestan.pillonel@chuv.ch (T.P.); gilbert.greub@chuv.ch (G.G.); 7 Institute of Medical Virology, University of Zurich, 8057 Zurich, Switzerland; huber.michael@virology.uzh.ch (M.H.); trkola.alexandra@virology.uzh.ch (A.T.); 8 Institute for Infectious Diseases, University of Bern, 3012 Bern, Switzerland; alban.ramette@ifik.unibe.ch (A.R.); christian.baumann@ifik.unibe.ch (C.B.); michel.koch@ifik.unibe.ch (M.C.K.); pascal.bittel@ifik.unibe.ch (P.B.); franziska.suter@ifik.unibe.ch (F.S.-R.); stephen.leib@ifik.unibe.ch (S.L.L.); 9 Department of Biosystems Science and Engineering, ETH Zurich, 4058 Basel, Switzerland; chaoran.chen@bsse.ethz.ch (C.C.); sarah.nadeau@bsse.ethz.ch (S.N.); 10 Center for Laboratory Medicine, 9001 Saint Gall, Switzerland; Yannick.Gerth@zlmsg.ch (Y.G.); Oliver.Nolte@zlmsg.ch (O.N.); 11 Federal Office of Public Health FOPH, 3097 Berne, Switzerland; tobias.schuster@bag.admin.ch (T.S.); Michael.Bel@bag.admin.ch (M.B.); 12 Spiez Laboratory, Federal Office for Civil Protection FOCP, 3700 Spiez, Switzerland; Cesar.Metzger@babs.admin.ch (C.M.J.A.M.); Jonas.Sieber@babs.admin.ch (J.S.); 13 Clinical Microbiology, Labormedizinisches Zentrum Dr. Risch, 9470 Buchs SG, Switzerland; nadia.wohlwend@risch.ch (N.W.); martin.risch@risch.ch (M.R.); lorenz.risch@risch.ch (L.R.); 14 Clinical Virology, University Hospital Basel, 4031 Basel, Switzerland; karoline.leuzinger@usb.ch (K.L.); hans.hirsch@unibas.ch (H.H.H.); 15 Transplantation & Clinical Virology, Department of Biomedicine, University of Basel, 4031 Basel, Switzerland; 16 Bioanalytica AG, 6006 Lucerne, Switzerland; Livia.Berlinger@bioanalytica.ch; 17 Viollier AG, 4123 Allschwil, Switzerland; christiane.beckmann@viollier.ch (C.B.); christoph.noppen@viollier.c (C.N.); maurice.redondo@viollier.ch (M.R.); 18 Rothen AG, 4002 Basel, Switzerland; i.steffen@labor-rothen.ch; 19 ADMED Microbiology, 2300 La Chaux-de-Fonds, Switzerland; Reto.Lienhard@ne.ch; 20 Coordination Commission of Clinical Microbiology, Swiss Society of Microbiology, 1033 Cheseaux, Switzerland; gladys.martinettilucchini@eoc.ch; 21 EOC Microbiological Laboratory, 6500 Bellinzona, Switzerland; 22 Institute of Social and Preventive Medicine, University of Bern, 3012 Bern, Switzerland; emmahodcroft@gmail.com; 23 Biozentrum, University of Basel, 4056 Basel, Switzerland; 24 Division of Infectious Diseases and Hospital Epidemiology, University Hospital Basel, 4031 Basel, Switzerland; 25 Faculty of Medical Sciences, Private University of the Principality of Liechtenstein, 9495 Triesen, Liechtenstein; 26 Centre of Laboratory Medicine, University Institute of Clinical Chemistry, University of Bern, 3010 Bern, Switzerland

**Keywords:** SARS-CoV-2, COVID-19, sequencing, surveillance, variant, mutation, N501Y, Switzerland, molecular epidemiology

## Abstract

The rapid spread of the SARS-CoV-2 lineages B.1.1.7 (N501Y.V1) throughout the UK, B.1.351 (N501Y.V2) in South Africa, and P.1 (B.1.1.28.1; N501Y.V3) in Brazil has led to the definition of variants of concern (VoCs) and recommendations for lineage specific surveillance. In Switzerland, during the last weeks of December 2020, we established a nationwide screening protocol across multiple laboratories, focusing first on epidemiological and microbiological definitions. In January 2021, we validated and implemented an N501Y-specific PCR to rapidly screen for VoCs, which are then confirmed using amplicon sequencing or whole genome sequencing (WGS). A total of 13,387 VoCs have been identified since the detection of the first Swiss case in October 2020, with 4194 being B.1.1.7, 172 B.1.351, and 7 P.1. The remaining 9014 cases of VoCs have been described without further lineage specification. Overall, all diagnostic centers reported a rapid increase of the percentage of detected VOCs, with a range of 6 to 46% between 25 to 31 of January 2021 increasing towards 41 to 82% between 22 to 28 of February. A total of 739 N501Y positive genomes were analysed and show a broad range of introduction events to Switzerland. In this paper, we describe the nationwide coordination and implementation process across laboratories, public health institutions, and researchers, the first results of our N501Y-specific variant screening, and the phylogenetic analysis of all available WGS data in Switzerland, that together identified the early introduction events and subsequent community spreading of the VoCs.

## 1. Introduction

Since December 2020, three emerging SARS-CoV-2 lineages—B.1.1.7 (N501Y.V1), B.1.351 (N501Y.V2), and P.1 (B.1.1.28.1; N501Y.V3)—have generated concern in public and scientific communities. All three lineages show a rapid spread and displacement of locally established SARS-CoV-2 lineages, in the United Kingdom (UK), South Africa (ZA), and Brazil (BR), respectively, where they were first detected [1,2,3,4,5,6,7,8]. The B.1.1.7 and B.1.351 lineages have subsequently been reported in many countries around the globe, including Switzerland. Most recently the P.1 lineage, exhibiting the N501Y and E484K mutations, among others, was described in Brazil [9,10,11] and has also been found in Japan [12]. It is hypothesized that the viral variants B.1.1.7, B.1.351, and P.1 are more transmissible compared to other circulating variants, due to a higher affinity towards the angiotensin-converting enzyme 2 (ACE2) receptor resulting from the N501Y mutation [13] and were defined as variants of concern (VoC). In the last week of December 2020, the B.1.1.7 lineage accounted for more than 25% of overall published genomes from the UK (according to the Global Initiative on Sharing Avian Influenza Data (GISAID) as of 19 January 2021), but it is estimated to account for up to 70% of transmission events in specific areas of the UK [14]. Waste-water screening in Switzerland suggests that the B.1.1.7 lineage was present in Switzerland in early December [15]. In South Africa, no reliable prevalence data on the B.1.351 lineage is available, but published data suggests that this VoC is also spreading more rapidly [6,16].

The first genome belonging to the B.1.1.7 lineage was detected in September 2020 in the UK (according to the GISAID database) and showed 17 lineage specific polymorphisms, eight of which are located in the 1273 amino acid spike glycoprotein (nucleotide position 21,563 to 25,384, [17,18,19] Table S1). The spike glycoprotein is crucial for viral infection of host cells and is an important target for neutralizing antibodies [20]. Some of the B.1.1.7 polymorphisms may modulate the protein’s function, such as the N501Y mutation in the receptor binding domain, the HV 69–70 deletion, and the P681H mutation in the furin cleavage site [21,22]. The HV 69–70 deletion at nucleotide position 21765–21770 of the SARS-CoV-2 genome results in a dropout of the spike glycoprotein (S) gene diagnostic target in some commercial PCR assays. Although the S gene dropout is not specific for the B.1.1.7 lineage, it may nevertheless be a good first approach to screen for B.1.1.7 variants [23,24]. This HV 69–70 deletion in the spike glycoprotein might favor immune escape [17]. The B.1.1.7 variant also carries several lineage specific mutations in the ORF8 gene (Table S1), which might also be associated with decreased host immunity against SARS-CoV-2. Indeed, the ORF8 protein disrupts antigen presentation and reduces the recognition and the elimination of virus-infected cells by cytotoxic T-cells [25].

The B.1.351 lineage was first detected in October 2020 in ZA (according to the GISAID database) and also shares the N501Y mutation, but has otherwise different lineage-determining polymorphisms (Table S1) and does not show a characteristic S gene dropout due to lack of the HV 69–70 deletion. Of particular concern is the spike glycoprotein E484K mutation, which has been shown to reduce binding affinities towards neutralizing antibodies [6,26,27]. Current administered vaccines in Switzerland include the Pfizer/BioNTech (since 19 December 2020) and the Moderna vaccines (since 12 January 2021). The overall rate of administered vaccines in Switzerland by the observational period was overall low: on 1 of February 2021 only 3.73 of 100 inhabitants were vaccinated and on 1 March 2021 9.47 of 100 inhabitants (https://www.covid19.admin.ch/en/epidemiologic/vacc-doses, accessed on 3 March 2021). In addition, the N484K mutants remain rare and we do not observe a strong selection pressure towards these variants. Some of the polymorphisms that the viral variants described here possess are also present in other SARS-CoV-2 lineages (Table S2) and hence raise the question about how viral variants and lineages evolve (parallelism or same ancestral strain) and what selective pressures are important at single patient and population levels. The origins of B.1.1.7 and B.1.351 remain speculative, but may include mutations during chronic infection in immunosuppressed patients exposed to convalescent plasma or other therapies [28], or potential recombination events between different lineages. As SARS-CoV-2 whole genome sequencing (WGS) is not performed uniformly across the globe, there may be other, unsampled, lineages also showing similar features of selection. Some variants may have been selected in intensive mink farms, where large outbreaks have been documented, as well as common cross-species transmission from human to minks and back [29]. The adaptations in VoCs may lead to a substantially higher case burden [5], potentially paving the way for additional waves of the pandemic, and continued challenge for healthcare systems across European countries. Therefore, rapid identification of the B.1.1.7, B.1.351, and P.1 lineages is very important, and should trigger intensified contact tracing, targeted public health interventions in affected geographical areas, and re-allocation of vaccination strategies to areas with increasing community transmission of the VoCs.

During December 2020, awareness of the B.1.1.7 and B.1.351 lineages and the epidemiological situations in the UK and ZA reached the public, while at the same time approximately 10,000 tourists from endemic areas arrived in Switzerland for ski holidays. In order to understand the spread of VoC and to adapt public health interventions accordingly, a multi-step screening concept was developed across diagnostic and research laboratories in collaboration with the Federal Office of Public Health (FOPH), the Spiez Laboratory from the Federal Office for Civil Protection (FOCP), the Coordination Commission of Clinical Microbiology of the Swiss Society of Microbiology (CCCM-SSM), and the National Reference Center for Emerging Viral Infections at the University Hospital Geneva. In this article, we share our experience of a nationwide screening strategy, its implementation, and early results on the spread of the VoCs in Switzerland.

## 2. Materials and Methods

Ethical statement. This study was conducted in close collaboration with the FOPH and was part of an epidemiological assessment (Communicable Diseases Legislation—Epidemics Act). In addition, the study was approved as a multi-center study by the leading ethical committee (Ethik Kommission Nordwest-und Zentralschweiz, EKNZ; Approval number 2019-01291).

**Figure 1:**
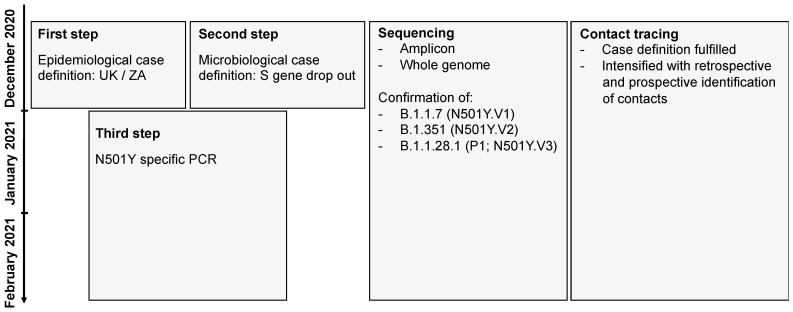
Diagnostic strategy to detect the B.1.1.7 and the B.1.351 in Switzerland. The flowchart shows the three-step strategy with (i) initial epidemiological case definitions with travels from the UK or South Africa, (ii) the diagnostic evidence due to a S gene dropout and (iii) the final establishment of a N501Y-specific PCR. In all steps amplicon based and whole genome sequencing was used to determine and confirm the lineage allocation.

Development of a screening strategy. Due to the highly probable introduction of the B.1.1.7 and B.1.351 lineages into the Swiss population, the FOPH, the Spiez Laboratory (within the FOCP), the CCCM-SSM, the National Reference Center for Emerging Viral Infections, and the diagnostic laboratories developed a pragmatic screening strategy for the VoC (Figure 1). The goal was to use already established infrastructures and reporting systems. The concept was communicated to cantonal physicians and diagnostic laboratories via the FOPH and FOCP and on the website of the CCCM-SSM [30]. Suspected and confirmed VoCs were reported to the FOPH and cantonal physicians, initiating extensive backward and forward contact tracing with the goal of rapidly interrupting transmission chains. The screening strategy was continuously adapted: a first step included an epidemiological case definition with a recent travel history to the UK or ZA, a second step included a microbiological case definition with an S gene dropout in the TaqPath™ COVID-19 Combo Kit diagnostic assay (Thermo Fisher), and a third step included the implementation of a N501Y-specific PCR. Further details of the establishment of the nationwide surveillance are provided in the supplementary part of this paper.

Included samples for sequencing and reporting. The initial identified samples, from 22 December 2020, were strongly biased towards the epidemiological and microbiological case definition (S gene dropout). From the beginning of January 2021, an increasing number of laboratories have joined the incentive and implemented N501Y-specific protocols. Meanwhile, older samples collected from September to December 2020 have also been sequenced. All VoCs were reported to the FOPH via an electronic reporting form.

Sanger sequencing protocols. For the sake of rapidity, amplicon-based sequencing, focusing on the S gene, was established at the National Reference Center for Emerging Viral Infections (HUG, Virology Laboratory) and implemented by other laboratories. The detailed protocols are available online [31]. In addition, amplicon-based sequencing focusing on the *ORF8* gene was performed at the Institute of Microbiology of the University Hospital Lausanne. Briefly, specific primers were used to generate an amplicon for Sanger sequencing. All sequences were then compared to available sequences on GISAID.

Whole genome sequencing protocols. For this study whole genome sequencing data were produced using Illumina and Oxford Nanopore Technologies (ONT, Oxford, UK) sequencing. SARS-CoV-2 genomes were generally amplified following the amplicon sequencing strategy of the ARTIC protocol (https://artic.network/ncov-2019, accessed on 3 March 2021) with V.1 or V.3 primers and 150 nucleotide paired-end sequenced, on an Illumina platform e.g., [32,33]. Most laboratories used Illumina based library preparations (NexteraXT or Nextera Flex). At the University Hospital Lausanne, libraries were prepared using the CleanPlex 5 SARS-CoV-2 Panel (Paragon Genomics). Further technical details of different sequencing protocols have been published [32].

A typical Nanopore sequencing library consisted of the pooling of PCR amplicons generated according to the ARTIC v3 protocol (https://artic.network/ncov-2019, accessed on 3 March 2021), which generates 400 bp amplicons that overlap by approximately 20 bp. Library preparation was performed with SQK-LSK109 (ONT) according to the ONT “PCR tiling of COVID-19 virus” (version: PTC_9096_v109_revE_06Feb2020, last update: 26 March 2020). Reagents, quality control and flow cell preparation were as described previously [34,35]. ONT sequencing was performed on a GridION X5 instrument (Oxford Nanopore Technologies) with real-time base calling enabled (ont-guppy-for-gridion v.4.2.3; fast base calling mode). Sequencing runs were terminated after production of at least 100,000 reads per sample. Bioinformatic analyses followed the workflow described (https://artic.network/ncov-2019/ncov2019-bioinformatics-sop.html, accessed on 3 March 2021) using artic version 1.1.3. Consensus sequences were generated using medaka (https://github.com/nanoporetech/medaka, accessed on 3 March 2021) and bcftools [36].

Each center used individual bioinformatic pipelines to check for sequencing quality and generate the consensus sequences details shared in GISAID (e.g., [32]; https://gitlab.com/RKIBioinformaticsPipelines/ncov_minipipe/, accessed on 3 March 2021). The consensus sequence data were either directly shared between diagnostic laboratories or via GISAID.

Phylogenetic inference. Global sequences and metadata were downloaded from GISAID [37,38] (as of 1 March 2021; 612,258 consensus sequences). Sequences with more than 10 percent Ns (133,229) and with incomplete dates (28,363) were removed. 456,204 sequences remained. The total dataset contained a total of 260 whole genomes from variants of concern with S:N501Y mutations from Switzerland (B.1.1.7, n = 675; B.1.351, n = 53; P.1, n = 11) (Table S4). The latest collection date of a N501Y positive whole genome dates to 11 February 2021, and the earliest to 30 November 2020 (accessed GISAID on 1 March). We inferred a time-calibrated phylogeny rooted to the first cases in Wuhan, China from December 2019, using a subset of global genomes and focal Swiss sequences. For sub-setting, we included 600 genomes evenly subsampled over canton (administrative subdivision), month and year for the focal area Switzerland; 15 genomes per country and per month in Europe, and 8 genomes per country and per month for the rest of the world (contextual samples) totaling 10,316 genomes, using the nextstrain software v.2.0.0.post1 (nextstrain.org) and augur v.10.3.8 [39]. The resulting alignment of focal and contextual genomes was used to infer clusters with zero single nucleotide mutations (SNPs) using a custom python script (https://github.com/appliedmicrobiologyresearch, accessed on 10 March 2021). Identified clusters were investigated regarding cantonal origin of the sample as well as known travel history.

## 3. Results

### 3.1. SARS-CoV-2 Case Numbers and Spatio-Temporal Distribution in Switzerland

The first cases of the B.1.1.7 lineage in Switzerland were detected in retrospect. Examined and sequenced samples from UK travel returners dated back to mid-October 2020 in Geneva and Lausanne and end of November 2020 in Basel. The first cases of B.1.351 were discovered in December 2020 in Schwyz (GISAID ID Switzerland/SZ-ETHZ-410256/2020 and Switzerland/BS-UHB-11011756/2020) who were travel returnees from ZA.

**Figure 2:**
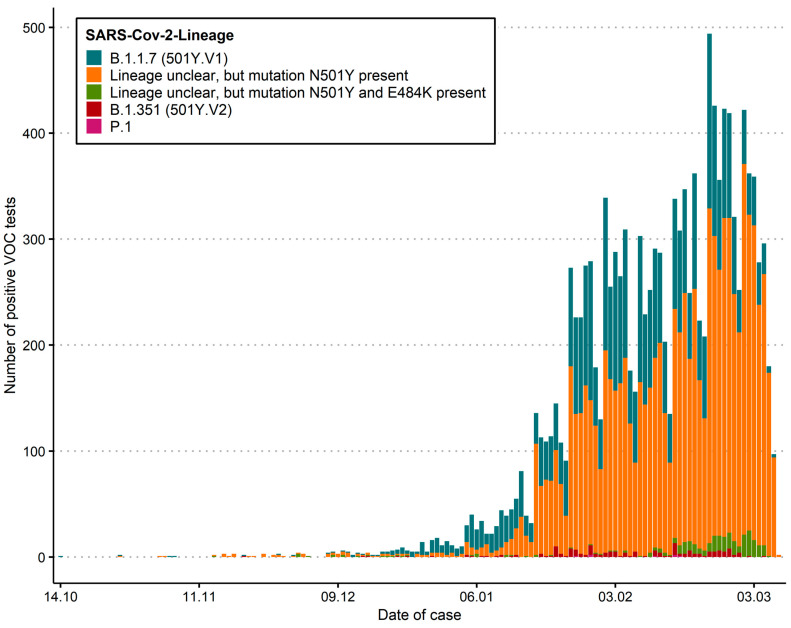
Distribution of absolute numbers as epidemiological curves across Switzerland. solute numbers reflect a biased sample set due to the initial case definitions, higher usage of antigen test in some regions, and distribution of diagnostic capacities. This does not reflect the prevalence of cases. The current number of specific lineages is also biased due to different sequencing capacities. “Lineage unclear” includes isolates which show a N501Y mutation, but where the lineage could not be determined by sequencing, either due to technical difficulties or due to non-availability of the sample.

Using our screening approach, a total of 13,387 samples carrying VoC have been found across different geographical regions (cantons, administrative subdivisions) (Table 1; Figure 2; Figure S1) from 14 October 2020 to 28 February 2021. 4373 of 13,387 (32.7%) could be confirmed by amplicon sequencing or whole genome sequencing, for which lineages were identified. 95.9% of these successfully sequenced genomes were assigned to the B.1.1.7 lineage. 3.9% were assigned to the B.1.351 lineage and 0.2% to the P.1 lineage.

Since the second week of January 2021, increasing numbers of SARS-CoV-2 positive samples were analysed using an N501Y-specific PCR. However, at this stage our data does not allow the reliable determination of a Swiss-wide prevalence, as not all PCR positive cases are fully re-analyzed with the N501Y-specific PCR. However, some laboratories re-analyze every SARS-CoV-2 positive case and thereby individual prevalence rates for VoCs could be determined for the last five weeks, clearly demonstrating the rapid increase and displacement of non-N501Y lineage strains (Table 2).

Some laboratories have reported the median age (with interquartile ranges) in years between patients with and without the N501Y variants. At the University Hospital Basel the median age of patients with N501Y positive was 34 years (IQR 12–47) whereas the median age of patients with N501Y negative was 38 years (27–54); at the University of Bern the media age was 33 years (IQR 20–51; N501Y positive) vs. 44 years (IQR 29–60; N501Y negative); at Bioanalytica the median age was 43 years (IQR 29–53; N501Y positive) vs. 48 years (IQR 32–67; N501Y negative); and at Viollier AG the median age was 41 years (IQR 26–54; N501Y positive) vs. 41 years (IQR 25–57; N501Y negative). This data may also be biased due to the fact that certain laboratories may receive samples more predominantly from pediatric physicians or hospitals.

### 3.2. Phylogenetic Relatedness of First Cases

**Figure 3:**
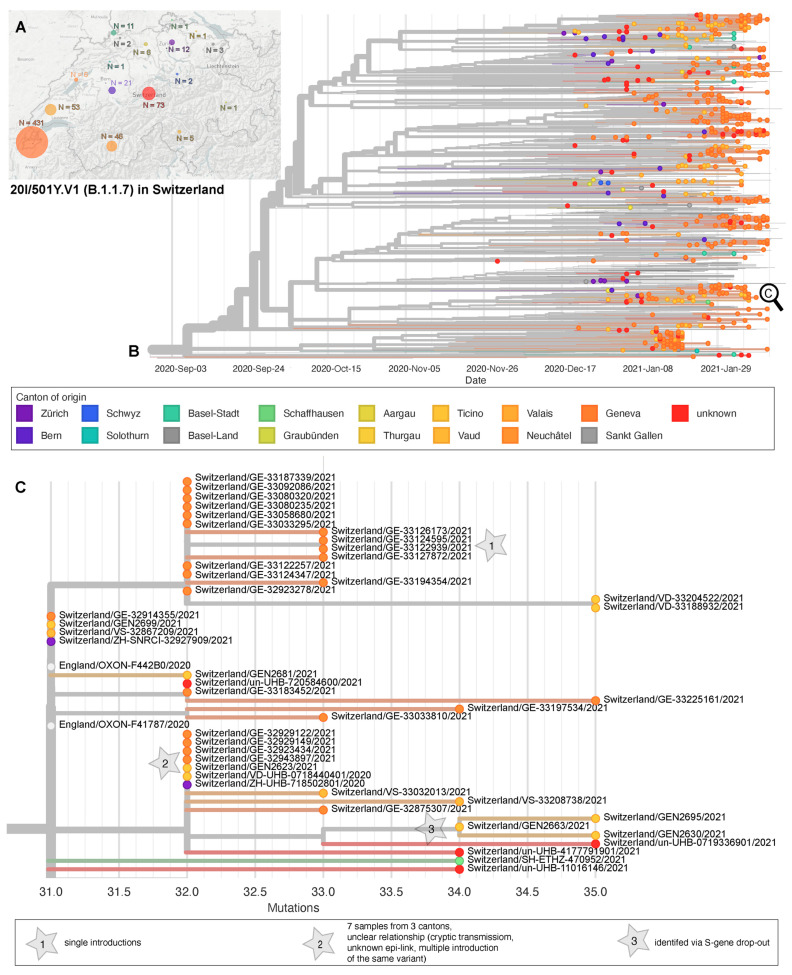
Phylogeny of sequenced B.1.1.7 cases in Switzerland. (**A**) Geographic distribution through Switzerland and (**B**) phylogenetic relationship of 675 genomes dating between 30 November 2020 and 11 February 2021 from the Geneva University Hospitals (*n* = 574), University Hospital Lausanne (*n* = 44), University Hospital Basel (*n* = 70), University of Bern (n = 10), University of Zurich (*n* = 14), and ETH Zurich (*n* = 20), Center for Laboratory Medicine St. Gallen (*n* = 1), Philip Morris International (*n* = 6). X-Axis scales to time. (**C**) Zoom into an exemplar, possible cryptic transmissions within a cluster, and single introductions, scale by mutations distance to the reference Wuhan/Hu1-1.

A total of 739 S:N501Y-carrying (B.1.1.7 n = 675, and B.1.351 n = 53; P.1 n = 11) Swiss high quality genomes were available for phylogenetic analysis. For six cases (known for University Hospitals Basel and Lausanne) a travel history to an endemic country or known contact to a traveler was available; however, for most cases the risk exposure was not available. For 666 cases the canton of residence was known. Using a 0 SNP threshold, we infer 33 out of the 53 B.1.351 cases to be single introduction events. Additionally, we count four (0 SNPs distance) clusters, three being from GE (12, 2, and 4 genomes each) and one being from BS with two epidemiologically linked cases that trace back to a ZA travel returner and a transmission to a family member (Figure S2). The first P.1 whole genome included here dates to 27 January 2021 and was detected in Zürich. The 11 totally included P.1 cases fall into two clusters (four cases each) as well as in three single introductions (Figure S3). The phylogenetic analysis of B.1.1.7 cases shows at least 301 single introductions into 16 cantons (Figure 3A), 291 without immediate links (0 SNP distance) to other genomes in the sub-sampled global dataset, three of which were known risk contacts or travelers (Figure 3B). Ten further single introductions had genetic links to genomes from UK samples, zero of which had known travel history or risk contact. We identified 93 clusters (0 SNP distance) comprising 356 (range 2–13) genomes. One cluster contained samples with a known travel link to the UK or risk contacts. Of interest, ten of these clusters contained cases from different cantons—suggesting outside of household transmission (Figure 3C).

## 4. Discussion

The epidemiological situation with the SARS-CoV-2 lineages is variable [40] and the emergence B.1.1.7 and B.1.351 in the UK and ZA resulted in the definition of so called variants of concern (VoC). The European Center for Disease Prevention and Control (ECDC) and the World Health Organization (WHO) strongly recommend identifying the viral lineages in order to monitor the distribution of VoCs, using sequencing for surveillance [41]. In Switzerland, we started a targeted screening program for VoC in December 2020, and are currently developing an unbiased sequencing-based surveillance program for new variants. Interestingly, we found the first case of the B.1.1.7 lineage from October 2020, by sequencing archived sample collections. However, in two large sets of 549 and 1511 SARS-CoV-2 samples from mid- to late-December 2020, we detected only sporadic cases of VoCs, suggesting that until then introductions and spread were not extensive. We calculate that the overall prevalence in Switzerland was less than 1% until the end of December 2020. Since then, our screening strategy showed a continuous increase of absolute case numbers, starting to ramp up in January 2021. This followed the Christmas holidays with thousands of ski tourists from endemic areas sojourning in Swiss ski resorts. During the first wave of the pandemic in 2020, skiing and associated activities resulted in a European wide spread of specific mutants linked to the alpine village Ischgl in Austria [42,43]. Similar, we have detected cases linked to a potential super spreading event in the ski resort of Wengen in December 2020, as reported in public news articles. Due to the concerns about high transmissibility of VoC, travelers from the UK and ZA were added to the quarantine list during the last week of December 2020. The comparison of our WGS data with a global dataset suggests direct links to the UK and indicates that a substantial number of the cases in January 2021 were due to individual introduction events from the UK. Unfortunately, information on links to UK travel are incomplete. Our experience suggests that detailed epidemiological data should be asked for and made available (such as travel history, contact to other infected people, etc.) In the future, more resources should be dedicated to this purpose. The identification of identical genomes from samples collected in distant regions of the country might indicate that cryptic transmission events were already happening, as only a few cases of household transmission were documented. However, they could also be due to multiple introductions of identical variants from the UK to Switzerland and this is a possibility that we cannot rule out.

Our sampling strategy focusing first on the epidemiological risk and a microbiological case definition including the S gene dropout, and the initial lack of diagnostic capabilities to confirm the VoC introduced a strong selection bias in the samples. Thus, our findings should be interpreted with care. Currently, our data does not allow us to properly determine the prevalence of VoCs in Switzerland. However, some laboratories have established a workflow including N501Y-specific PCR for all SARS-CoV-2 positive cases—this allowed us to monitor the increase in prevalence across tested samples in these individual laboratories. Available prevalence data from surveillance efforts aiming at capturing the prevalence of VOCs is visualized (https://ibz-shiny.ethz.ch/covidDashboard/variant-plot/index.html and https://ispmbern.github.io/covid-19/variants, accessed on 3 March 2021). Nevertheless, our pragmatic approach indicates that, prior to the Christmas holidays, the VoC distribution in Switzerland was low (<1%) and now has reached within 8 weeks likely rates of 20%–45%. Our phylogenetic analysis provides important evidence that community transmission started to increase after New Year and continuously accelerated in mid-January 2021.

VoC identification based on the S gene dropout is not specific and sensitive enough to identify VoC lineages (see Table S1). Our WGS data of samples collected in December 2020 showed that most of the S gene dropout samples were due to the B.1.258 lineage, at least in eastern Switzerland. The distribution and details of the B.1.258 lineage is further described on nextstrain.org, https://cov-lineages.org/lineages/lineage_B.1.258.html and https://covariants.org/variants/S.N439K (accessed on 3 March 2021). In our global phylogenetic tree, all B.1.258 are monophyletic, as they should be per definition of PANGO lineages. The lineage occurs mostly in European countries. Due to this geographical distribution pattern, the curious phenomenon emerged that in western Switzerland the S dropout screen was an efficient approach to detect the B.1.1.7. lineage, whereas in eastern Switzerland it was confounded by other more prevalent lineages. For this reason, the pre-test probability for the B.1.1.7 lineage using the S dropout was different, based on the local epidemiology.

The B.1.351 and P.1 lineages cannot be identified based on the S gene dropout. VoC can be confirmed using either amplicon based sequencing of the S gene or whole genome sequencing. Both sequencing approaches show specific advantages and disadvantages in terms of speed, costs, and resolution for molecular epidemiological studies. These aspects have to be carefully evaluated when establishing a screening program. Mixed usage may allow the strength of both methods. In order to efficiently select samples for subsequent sequencing, we have implemented a N501Y-specific PCR in many laboratories throughout Switzerland. The challenge, as most diagnostic institutions were focusing on high throughput testing using fully automated robotic systems, was to re-establish a manual method including separate RNA extraction. It took several weeks to establish the workflows in larger laboratories and to implement new variables for reporting. Similar to the UK, our data shows an increase in positivity rates across time and the B.1.1.7 lineage displaces other circulating strains. Continues surveillance of circulating strains is critical to gain critical knowledge on the transmission of new variants [44,45].

## 5. Conclusions

Surveillance of these and other VoCs may become more important with new selective pressures such as therapeutics and vaccination. The Swiss model, to monitor in a first phase with epidemiological and microbiological case definitions and in a second phase with a specific PCR, allowed the rapid screening of isolates and identification of the N501Y mutation as a surrogate marker for a potentially more transmissible variant. The subsequent confirmation with sequencing provides an efficient way to rapidly identify certain VoCs. It is strongly recommended to further sequence the VoCs and not stop at the identification of the N501Y mutations. Lineage or whole genome resolution provides highly valuable information for public health management in the search for future upcoming variants such as vaccine escape mutants. It is clearly time for nations to seriously consider implementing national surveillance programs with an unbiased sequencing approach, incorporating sustainable elements for other key pathogens and potential future pandemics.

## Figures and Tables

**Table 1:** Absolute numbers of variants of concern (VoC) including cases of B.1.1.7, B.1.351, and P.1 in Switzerland and Principality of Liechtenstein. Absolute numbers reflect a biased sample set due to the initial case definitions and biased distribution of diagnostic capacities. The numbers and distributions of lineages is likely biased due to delay in processing and different sequencing capacities. Cantons: AG, Aargau; AI, Appenzell Innerrhoden; AR, Appenzell Ausserrhoden; BE, Bern; BL, Basellandschaft; BS, Basel-Stadt; FR, Fribourg; FL, Fürstentum Lichtenstein; GE, Geneva; GL, Glarus; GR, Graubünden; JU, Jura; LU, Lucerne; NE, Neuchatel; NW, Nidwalden; OW, Obwalden; SG, St. Gallen; SH, Schaffhausen; SO, Solothurn; SZ, Schwyz; TG, Thurgau; TI, Tessin; UR, Uri; VD, Vaud; VS, Vallais; ZG, Zug; ZH, Zürich.

Canton	B.1.1.7 (501Y.V1)	B.1.351 (501Y.V2)	P.1	Lineage Not Specified N501Y pos	Lineage Not Specified N501Y and E484K pos	VoC Total
AG	145	3		771	12	931
AI	1			3		4
AR		1		54		55
BE	528	29		442	33	1032
BL	259	5		133	1	398
BS	77	3		286		366
FR	289	9	1	168	61	528
FL	32	1		5	1	39
GE	406	11	2	1432		1851
GL	6	1	1	10	4	22
GR	175	2		243	4	424
JU	126	4		28		158
LU	24	1		336	42	403
NE	104			331		435
NW	3			34	7	44
OW				17		17
SG	146	31		492	6	675
SH	31	10		37	1	79
SO	271	3		141	2	417
SZ	38	4		124	8	174
TG	68	14	2	329		413
TI	157	2		239	15	413
UR	3			6	2	11
VD	726	20		910	9	1665
VS	199	1		440		640
ZG	8	1		130	10	149
ZH	372	16	1	1601	54	2044
CH/FL	4194	172	7	8742	272	13387

**Table 2:** Relative proportions of N501Y of total detected positive cases. This table shows the relative detection rate of the N501Y positive variants according to laboratory.

	25–31 January	1–7 February	8–14 February	15–21 February	22–28 February
Bioanalytica	6%	21.2%	31.1%	35.9%	40.5%
LMZ Risch	18.5%	24.8%	29%	48%	57%
University Hospital Basel	29.5%	49.3%	63.3%	50%	69.4%
University of Bern	10.2%	35.9%	30%	44.7%	57.7%
University Hospital Geneva	46.1%	61.3%	75.5%	67.0%	81.7%
University Hospital Lausanne	30.4%	51.5%	53.2%	65.4%	81.4%
University of Zurich	20.2%	34.5%	36.6%	46.7%	65.6%
Viollier	15%	23.6%	31.2%	38%	61.6%

## Data Availability

All sequencing data of this study is already available at GISAID.

## Supplementary Materials

The following materials and figures are available online at https://www.mdpi.com/2076-2607/9/4/677/s1, Figure S1: Absolute case numbers for selected cantons in Switzerland over time, Figure S2: Phylogeny of sequenced B.1.351 cases in Switzerland dating from 14 December 2020 to 11 February 2021, Figure S3: Phylogeny of sequenced P.1 cases in Switzerland dating from 27 January 2021 to 11 February 2021, Table S1: Mutations of the new SARS-CoV-2 lineages B.1.1.7, B.1.351 and P.1 (B.1.1.28.1), Table S2: N501Y mutations across different viral lineages since September 2020, Table S3: Screening methods used by different diagnostic laboratories as of 20 January 2021, Table S4: GISAID database identifier, Table S5: List of laboratories submitting to GISAID, supplementary methods and results.
