# Supplementary material for: SARS-CoV-2 N501Y Introductions and Transmissions in Switzerland from Beginning of October 2020 to February 2021—Implementation of Swiss-Wide Diagnostic Screening and Whole Genome Sequencing
Source: Microorganisms. 2021 Mar 25;9(4):677. doi: 10.3390/microorganisms9040677 (PMC8064472; doi:10.3390/microorganisms9040677)
Supplement: Supplementary file 1 [file microorganisms-09-00677-s001.zip › Supplementary Materials_N501Y_AE.docx]

**Supplementary Materials:**

**Figure S1.** Absolute case numbers for selected cantons in Switzerland over time. For each canton the individual cases of detected N501Y, E484K mutations or, if available, lineage allocation is shown.

**Figure S2.** Phylogeny of sequenced B.1.351 cases in Switzerland dating from 14^th^ December 2020 to 11^th^ February 2021. (A) Geographic distribution of cases. (B) Genomic divergence of cases. Known household transmission after travel return indicated by star. (C) Zoom into a larger cluster from Geneva with unknown epi-link.

**Figure S3.** Phylogeny of sequenced P.1 cases in Switzerland dating from 27^th^ January 2021 to 11^th^ February 2021. (A) Geographic distribution of cases. (B) Genomic divergence of cases.

**Table S1.** Mutations of the new SARS-CoV-2 lineages B.1.1.7, B.1.351 and P.1 (B.1.1.28.1). Data from Global Initiative on Sharing Avian Influenza Data (GISAID) and Covariants.org/shared-mutations. Full SARS-CoV-2 genome of Wuhan-Hu-1 is available (https://www.ncbi.nlm.nih.gov/nuccore/MN908947).

**Table S2.** N501Y mutations across different viral lineages since September 2020**.** Based on GISAID database (access 15.01.2021).

**Table S3.** Screening methods used by different diagnostic laboratories as of 20th January 2021**.** N501Y screening assay was most commonly used from TIB MOLBIOL (Germany), but also in-house developed assays were used. University centers offering whole genome sequencing included the University Hospital Basel (Division of Clinical Bacteriology and Mycology), University of Bern (Institute for Infectious Diseases), University Hospital Geneva (Virology Laboratory), University Hospital Lausanne (Institute of Microbiology), and the University of Zurich (Institute of Medical Virology). In addition, ETHZ (D-BSSE core facility) sequenced samples.

**Table S4.** GISAID database identifier**.** The N501Y-carrying genomes from Switzerland included into phylogenetic analysis are listed.

**Table S5.** List of laboratories submitting to GISAID**.** We thank all the laboratories actively sharing their datasets.

**Supplementary information.**

**Supplementary methods.**

Development of a screening strategy*.* As a first step, an epidemiological case definition with recent travel history to the UK or ZA was used to identify potential carriers of the VoC. In mid-January 2021 this was expanded to BR. Both direct and indirect contacts of people travelling from these areas were considered. Patient travel history was recorded on mandatory FOPH reporting forms by clinical and laboratory institutions [1], as well as by cantonal physicians during contact tracing. In Switzerland, quarantine upon arrival was made mandatory for travelers from the UK and ZA from 28th December 2020; and from BR from 21st January 2021 [2].

As a second step, a microbiological case definition was used. LZM Risch AG using the TaqPath™ COVID-19 Combo Kit diagnostic assay (Thermo Fisher) noted a significant increase in S gene dropouts through November and December 2020. This multi-target PCR assay target sequences within the SARS-CoV-2 genes *ORF1ab*, *N* and *S*. A geographical distinction was observed: S gene dropouts were mainly noted in the eastern region of Switzerland, whereas other laboratories using the same assay in the western region of Switzerland noted an increase after a delay of four weeks. The S gene dropout is explained by a deletion at the positions 21765–21770 (HV 69–70) and is an indication for the B.1.1.7 lineage, as well as other non-VoC lineages. For this reason, and because initially variant-specific PCR assays were not yet available, the sequencing of all S gene dropouts was strongly recommended. A similar approach was developed by Danish [3] and Portuguese colleagues [4]. However, during November and December, most of the S gene dropouts detected were from another emerging lineage, B.1.258, not showing the N501Y mutation, but featuring the HV 69–70 deletion and the N439K mutation [5].

During the Christmas holidays 2020, personnel and technical resources were limited, and focus on these first two steps provided an initial screening program (from 22nd December,). In January 2021, the screening strategy was modified with a third step: several N501Y-specific PCR protocols were validated and established in laboratories throughout the country. All diagnostic laboratories in Switzerland were contacted on 15^th^ January 2021 via the FOCP to encourage and establish a N501Y-specific PCR. The CCCM-SSM published concomitantly additional recommendations on the society website (www.swissmicrobiology.ch). Since then, 21 laboratories have started to validate and implement a N501Y-specific PCR (as of 02.02.2021).

Various laboratories have used different PCR approaches to increase the pre-test probability of the identification of VoC. **Table S3** summarizes the different PCR approaches used by centers until 20th of January (**Table S3**). Most centers used the commercial assay SARS Spike N501Y (53-0780-96; TIB MOLBIOL, Germany). In addition to the N501Y-specific PCR, at the University Hospital Lausanne, *ORF8* PCR/sequencing was used for the initial 12 samples received, in order to rapidly obtain results based on the presence/absence of the B.1.1.7 specific mutations C27972T, G28048T and A28111G [6], while waiting for the results of whole genome sequencing and the implementation of the S dropout and N501Y-specific PCR.

**Supplementary results.**

The initial S gene dropout based screening showed a large geographical heterogeneity in terms of detected B.1.1.7 variants. Whereas in the eastern parts of Switzerland the S gene dropout based screening resulted only in about 1% of identified B.1.1.7 variants, in the western parts of Switzerland the percentage of B1.1.7 variants within the S gene dropouts was much higher e.g., at the University Hospital Lausanne, as many as 78.9% (101 of 128).

This initial screening was implemented in a few centers in the last two weeks of December 2020, during which time there was a selection bias due to the case definition and limited sequencing capacity over the Christmas holidays. Complementary to these cases, two additional datasets were analysed using whole genome sequencing: the first included 545 randomly selected SARS-CoV-2 samples from throughout Switzerland (Viollier AG, a large private laboratory) from 18th to 24th December 2020. Within this first dataset, no VoC was found. A second set focused on 1511 samples showing the S gene dropout (LMZ Dr. Risch, a large private laboratory) from 21st to 27th December 2020. From a total of 1511 PCR positive cases (15.1% positivity rate), 137 (9.1%) samples showed an S gene dropout. 79 of 137 S gene dropout samples were sequenced - and in 6/79 (8%) a B.1.1.7 lineage was found. This corresponds to 0.06% of the overall SARS-CoV-2 positive cases.

References

1. Health FOoP. COVID-19 Reporting, 2021. Available online: https://www.bag.admin.ch/bag/de/home/krankheiten/infektionskrankheiten-bekaempfen/meldesysteme-infektionskrankheiten/meldepflichtige-ik/meldeformulare.html (accessed on 6 February 2021).
2. Health FOoP. Coronavirus: Entering Switzerland, 2021. Available online: https://www.bag.admin.ch/bag/en/home/krankheiten/ausbrueche-epidemien-pandemien/aktuelle-ausbrueche-epidemien/novel-cov/empfehlungen-fuer-reisende/quarantaene-einreisende.html (accessed on 6 February 2021).
3. Institut SS. Nye PCR-Tests Skal Styrke Kontrollen med Muterede Varianter. 2021. Available online: https://www.ssi.dk/aktuelt/nyheder/2021/nye-pcr-tests-skal-styrke-kontrollen-med-muterede-varianter (accessed on 6 February 2021).
4. Borges, V.; Sousa, C.; Menezes, L.; Gonçalves, A.M.; Picão, M.; Almeida, J.P.; Vieita, M.; Santos, R.; Silva, A.R.; Costa, M.; et al. Tracking SARS-CoV-2 VOC 202012/01 (lineage B.1.1.7) dissemination in Portugal: Insights from nationwide RT-PCR Spike gene drop out data. *Virological* **2021**. Available online: https://virological.org/t/tracking-sars-cov-2-voc-202012-01-lineage-b-1-1-7-dissemination-in-portugal-insights-from-nationwide-rt-pcr-spike-gene-drop-out-data/600 (accessed on 6 February 2021).
5. Hodcroft, E.B. CoVariants S:N439K. 2021. Available online: https://covariants.org/variants/S.N439K (accessed on 6 February 2021).
6. Voloch, C.M.; da Silva Francisco, R.; de Almeida, L.G.; Cardoso, C.C.; Brustolini, O.J.; Gerber, A.L.; Guimarães, A.P.D.C.; Mariani, D.; da Costa, R.M.; Ferreira, O.C.; et al. Preliminary genomic characterisation of an emergent SARS-CoV-2 lineage in the UK defined by a novel set of spike mutations. *Virological* **2020**. Available online: https://virological.org/t/preliminary-genomic-characterisation-of-an-emergent-sars-cov-2-lineage-in-the-uk-defined-by-a-novel-set-of-spike-mutations/563 (accessed on 6 February 2021).
