# Supplementary material for: SARS-CoV-2 N501Y Introductions and Transmissions in Switzerland from Beginning of October 2020 to February 2021—Implementation of Swiss-Wide Diagnostic Screening and Whole Genome Sequencing
Source: Microorganisms. 2021 Mar 25;9(4):677. doi: 10.3390/microorganisms9040677 (PMC8064472; doi:10.3390/microorganisms9040677)
Supplement: Supplementary file 1 [file microorganisms-09-00677-s001.zip › Table S2.docx]

**Table S2.** N501Y mutations across different viral lineages since September 2020**.** Based on GISAID database (access 15.01.2021).

| **Lineage** | **Frequency** | **Countries** | **Number genomes** | **Percentage of**  **N501Y** |
| --- | --- | --- | --- | --- |
| B.1 | 6 | USA, United Kingdom | 24057 | 0.025 |
| B.1.1 | 3 | United Kingdom, USA | 21672 | 0.014 |
| B.1.1.189 | 1 | Denmark | 113 | 0.885 |
| B.1.1.7 | 3072 | Australia, Canada, Denmark, United Kingdom, Finland, Hong Kong, India, Ireland, Israel, Italy, Netherlands, Norway, Portugal, Singapore, Spain | 3155 | 97.369 |
| B.1.1.70 | 484 | United Kingdom | 1009 | 47.968 |
| B.1.160 | 1 | United Kingdom | 4536 | 0.022 |
| B.1.177 | 6 | United Kingdom | 35019 | 0.017 |
| B.1.351 | 326 | United Kingdom, South Africa, Switzerland | 329 | 99.088 |
| B.1.5 | 9 | United Kingdom | 10533 | 0.085 |
| B.1.83 | 1 | United Kingdom | 25 | 4 |
