# Supplementary figures and images for: SARS-CoV-2 N501Y Introductions and Transmissions in Switzerland from Beginning of October 2020 to February 2021—Implementation of Swiss-Wide Diagnostic Screening and Whole Genome Sequencing
Source: Microorganisms. 2021 Mar 25;9(4):677. doi: 10.3390/microorganisms9040677 (PMC8064472; doi:10.3390/microorganisms9040677)

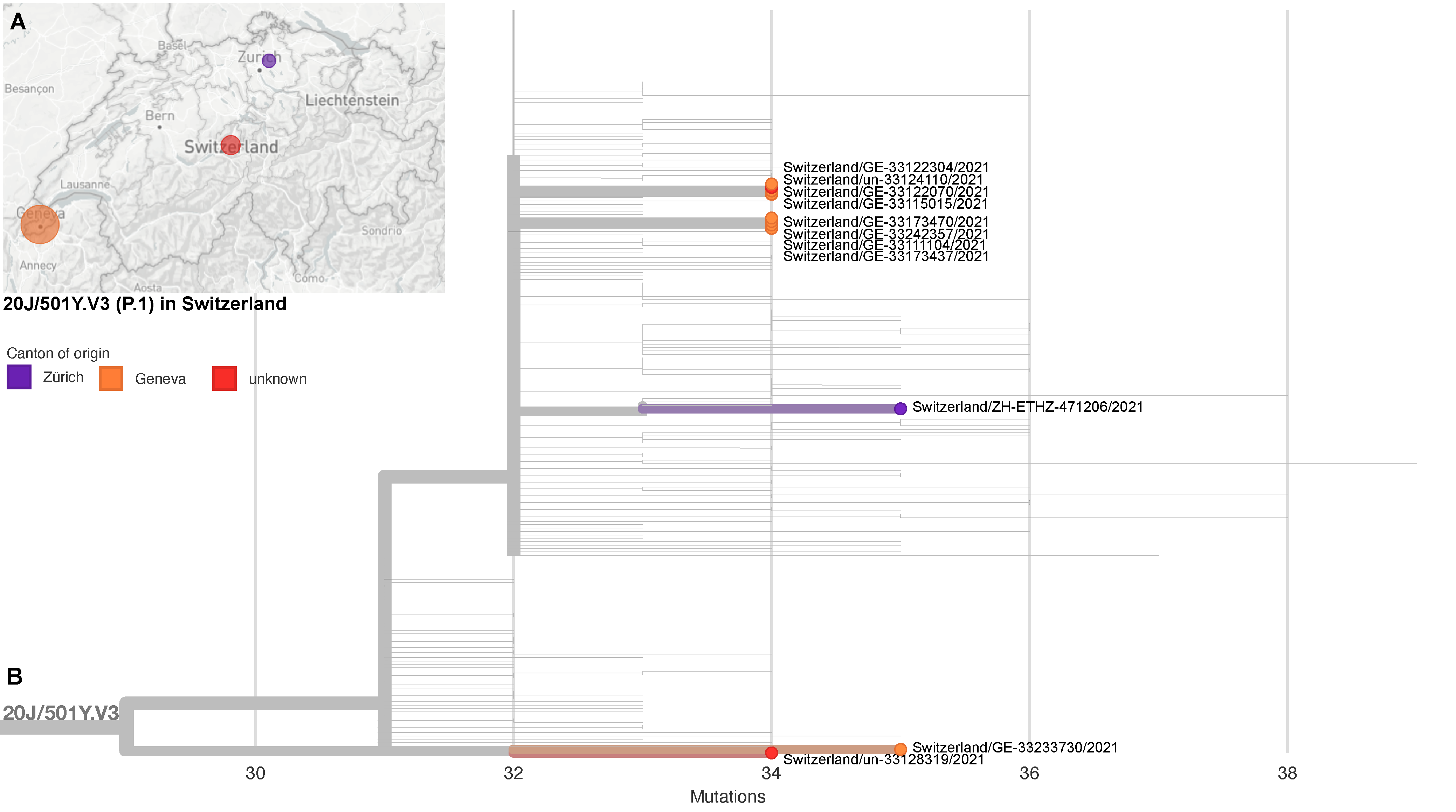

Supplement: Supplementary file 1 [file microorganisms-09-00677-s001.zip › Figure S3_20J-501Y.V3_nextstrain_ncov_switzerland-01.tif]

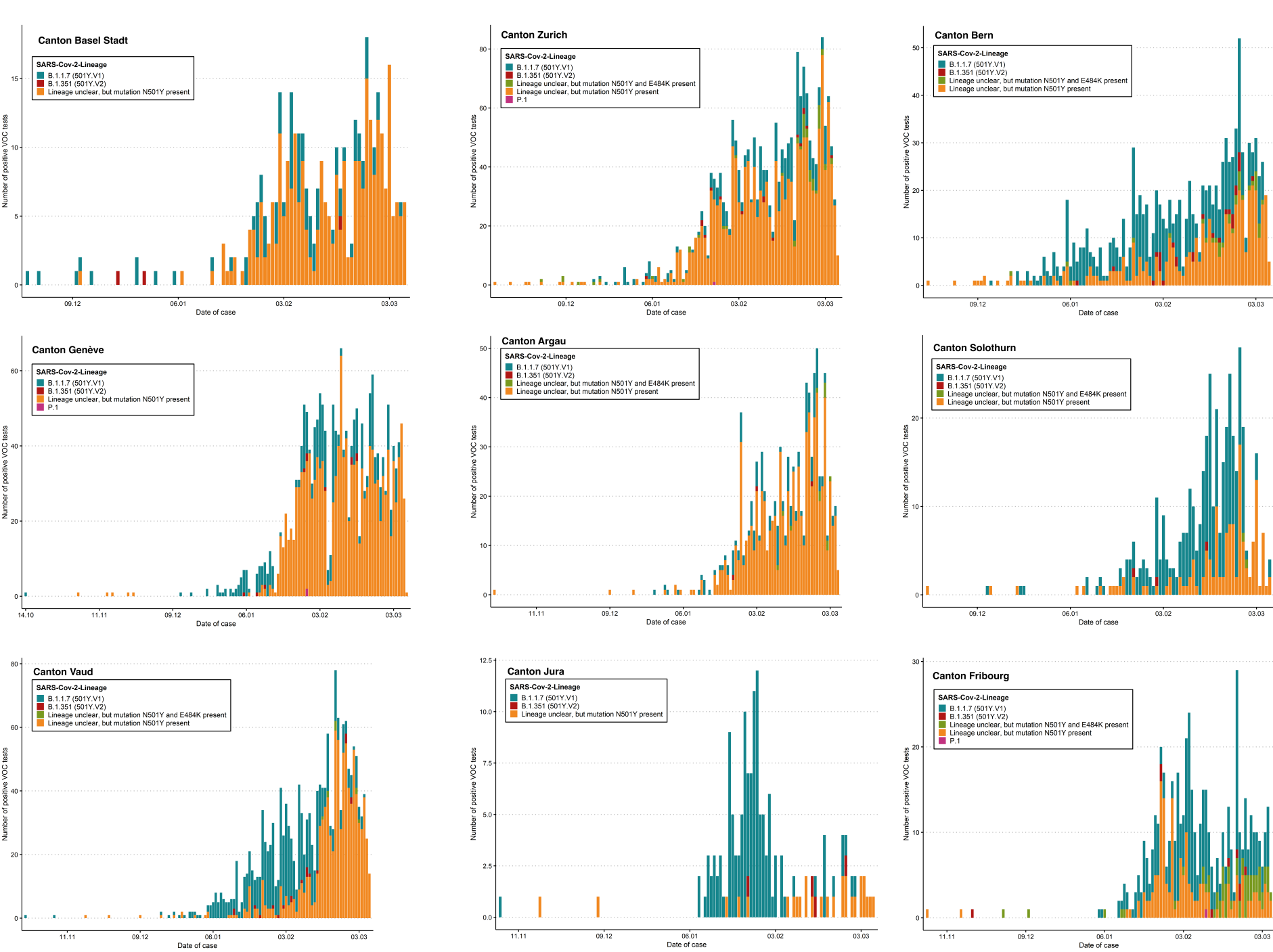

Supplement: Supplementary file 1 [file microorganisms-09-00677-s001.zip › Figure S1.pdf]

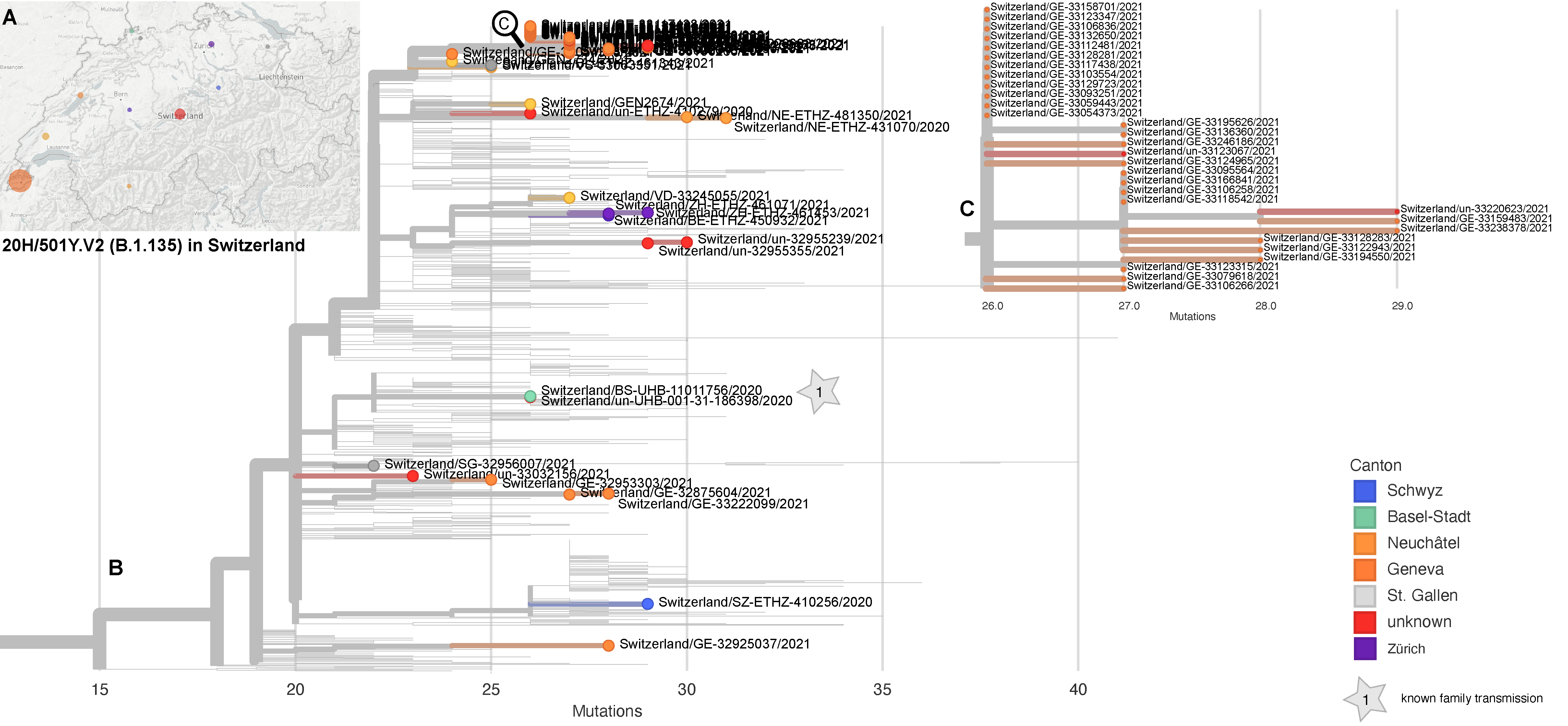

Supplement: Supplementary file 1 [file microorganisms-09-00677-s001.zip › Figure S2_20H-501Y.V2_mut_nextstrain_ncov_switzerland-01.tif]
